# Early Detection and Intervention of ASD: A European Overview

**DOI:** 10.3390/brainsci7120159

**Published:** 2017-12-01

**Authors:** María Magán-Maganto, Álvaro Bejarano-Martín, Clara Fernández-Alvarez, Antonio Narzisi, Patricia García-Primo, Rafal Kawa, Manuel Posada, Ricardo Canal-Bedia

**Affiliations:** 1 INICO, Instituto Universitario de Integración en la Comunidad, Universidad de Salamanca, 37005 Salamanca, Spain; mmmaria@usal.es (M.M.-M.); cjf2146@columbia.edu (C.F.-A); rcanal@usal.es (R.C.-B.); 2 IRCCS Stella Maris Foundation, 56018 Pisa, Italy; antonio.narzisi@fsm.unipi.it; 3 IIER, Instituto de Salud Carlos III, 28029 Madrid, Spain; PGARCIAPRIMO@externos.isciii.es (P.G.-P.); mposada@isciii.es (M.P.); 4 Faculty of Psychology, University of Warsaw, Stawki 5/7, 00-183 Warszawa, Poland; rkawa@psych.uw.edu.pl

**Keywords:** ASD, screening tools, detection, early intervention, Europe

## Abstract

Over the last several years there has been an increasing focus on early detection of Autism Spectrum Disorder (ASD), not only from the scientific field but also from professional associations and public health systems all across Europe. Not surprisingly, in order to offer better services and quality of life for both children with ASD and their families, different screening procedures and tools have been developed for early assessment and intervention. However, current evidence is needed for healthcare providers and policy makers to be able to implement specific measures and increase autism awareness in European communities. The general aim of this review is to address the latest and most relevant issues related to early detection and treatments. The specific objectives are (1) analyse the impact, describing advantages and drawbacks, of screening procedures based on standardized tests, surveillance programmes, or other observational measures; and (2) provide a European framework of early intervention programmes and practices and what has been learnt from implementing them in public or private settings. This analysis is then discussed and best practices are suggested to help professionals, health systems and policy makers to improve their local procedures or to develop new proposals for early detection and intervention programmes.

## 1. Introduction

Autistic Spectrum Disorders (ASD) are severe early-onset neurodevelopmental disorders characterized by significant difficulties in social communication, as well as restrictive interests and repetitive behaviours that critically affect daily living activities [1]. Over the last years there has been a very significant increase in ASD prevalence. Several prevalence studies conducted in the European context indicate rates reaching or exceeding 1% [2,3]. Other prevalence studies conducted in non-European regions have higher prevalence estimates ranging from 1.47% [4] to 2.64% [5]. Indeed, there is no doubt that the prevalence of ASD has steadily increased over the past 30 years [6]. Regardless of the possible causes for such increase, one of the consequences acknowledged internationally is that healthcare systems will have to be structured and organized accordingly, to address the needs of the growing population of children diagnosed with ASD. Therefore, there is an imperative necessity to know and reflect on current screening, diagnosis and treatment strategies, as a way to find effective solutions to guarantee that children with ASD and their families receive the required attention in order to achieve optimal treatment results.

An important factor in this reflection is that both parents and relatives experience the emergence of ASD symptoms in a child as a very worrying and usually destabilizing event of the personal and family well-being. Consequently, from that moment on a generally complex and long process of adaptation begins, in which they hastily have to face challenges such as receiving a formal diagnosis, accessing an appropriate treatment and adapting the family’s (and professional) dynamics to this new reality of autism in the family. Among the aspects that have been identified as contributors to this loss of well-being (besides individual factors associated with the characteristics of the children and their parents), there are contextual factors related to the social environment and availability of professional support services. For instance, one of the main sources of parental stress reported by parents of children with ASD is the lack of adequate professional support [7]. It has also been found that the delay in attending to the parents’ initial concerns, the diagnostic delay and not accompanying the diagnosis (even if it is early) with concrete guidelines for the intervention, increases the parents’ stress [8].

Despite the high and increasing prevalence of ASD and the personal and family costs of autism, these disorders have received little attention in the European public health services field (and in the rest of the world). The personal and family characteristics mentioned above have important implications in terms of planning and prioritizing healthcare, educational and social services. In order to promote the development of more appropriate strategies aimed at improving the response to autism, the European Parliament adopted in 2015 the Written Declaration on Autism, calling for a European strategy for autism that supports accurate detection and diagnosis across Europe and promote evidence-based interventions for children with autism. The Directorate-General for Health and Food Safety (DG Santé) of the European Commission agreed to fund Autism Spectrum Disorders in the European Union (ASDEU) project [9] whose efforts, within Work Package 2, are specifically aimed at promoting successful early detection and evidence-based early intervention for people with autism and their families in the European context.

Thus, the general objective of this literary review is to describe the different screening procedures, as well as early intervention programmes for ASD reported in scientific publications based on European experiences, placing more emphasis on contextual factors. As a specific objective, a description of the advantages and disadvantages of different screening experiences will be provided (both test-based screening programmes as well as developmental surveillance programmes and other observational measures for this purpose) with the ultimate purpose of examining their impact within the context in which they are applied. In addition, this review will also give account of the different programmes and practices of early intervention for ASD and will describe the different characteristics or knowledge that has been obtained from applying them in public or private settings.

This paper is structured as follows: Section 2 and Section 3 presents the screening programme topic and Section 4 and Section 5 the early intervention subject. A final Section 6 has been added to conclude with the integration of information about both topics. Section 2 introduces the methodology followed to review the screening programmes in the first paragraph and all the consequent text presents a narrative of the results obtained, accompanied by Table 1, Table 2 and Table 3, where all the information has been synthetized. In Section 3, the main strengths and limitations of the screening programmes have been discussed. Section 4 introduces the main results on early intervention programmes and Section 5 focuses on the analysis of European practices. The article concludes with a set of proposals for action aimed at professionals and researchers.

## 2. Description of the Early Detection ASD Experiences in Europe

**Figure 1:**
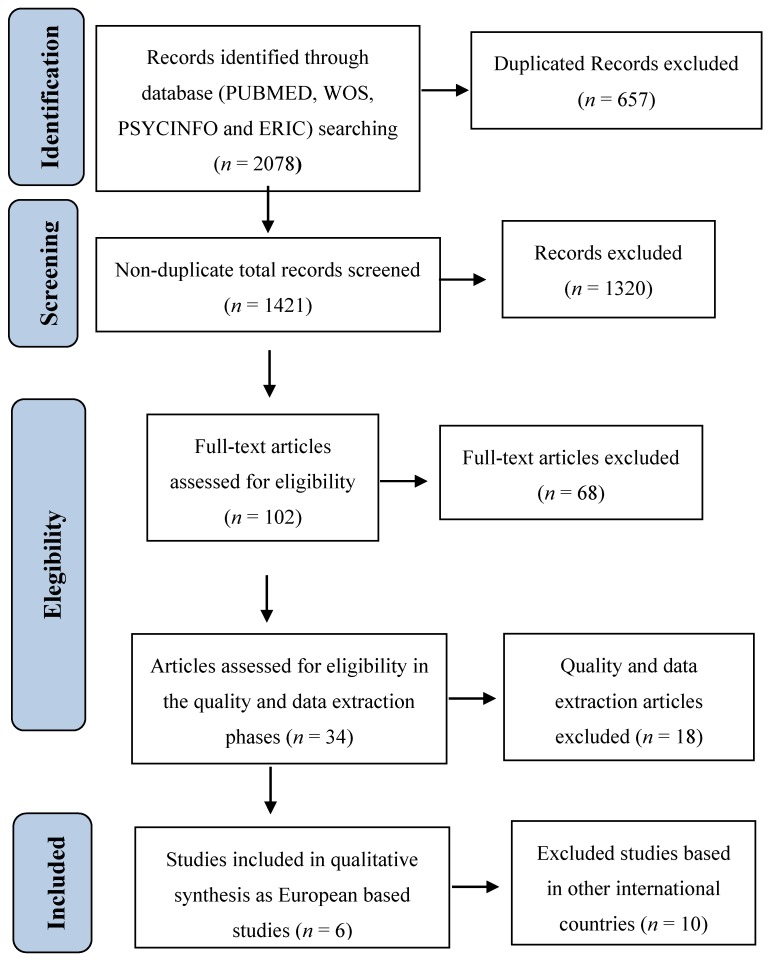
Preferred Reporting Items for Systematic Reviews and Meta-Analyses (PRISMA) flowchart representing the identification and selection of studies.

According to the research conducted under the ASDEU project [9] (see Table A1 in Appendix A for the search strategy), in Europe there are 6 studies (see Figure 1 for the study selection process and more details of the methodology protocol can be provided upon request) published in scientific English peer-review journals (to assure the replicability of the study) between January 1992 and April 2015, which describe in ample detail the scientific research on early screening for ASD and other neurodevelopmental disorders, more specifically for the age range of 14–36 months and targeting the general population (Level 1). These 6 detection references meet quality criteria, providing the necessary data to evaluate the effectiveness of the screening programme of each study. Other studies were excluded for the following reasons: (a) duplicate participant data in study sample; (b) did not include diagnostic evaluations supported by the scientific community as a gold standard for the confirmation of positive cases; or (c) did not include a summary of the diagnosis given to each case. After concluding the search process, the ASDEU researchers’ network was contacted to inquire about unpublished ongoing screening experiences or studies that were in the process of publication. As a result, a study that met the criteria mentioned above, carried out in France and recently published [10], was added to this review. In addition, another two on-going studies with unpublished data, one in Italy (Q-CHAT [11]) and another one in Spain (M-CHAT-R [12]), are currently being carried out. 

Table 1 shows a summary of the ASD early detection tools most frequently used in the European context and their most relevant characteristics. The Checklist for Autism in Toddlers (CHAT) [13,14] was the first tool that was applied in Europe and the only one originally developed in our continent. The work developed to create this tool has served as a conceptual and methodological basis for the development of other instruments subsequently created, which in some cases have achieved more satisfactory results when applied to the general population, such as Modified Checklist for Autism in Toddlers (M-CHAT) [15], the Early Screening Autistic Traits Questionnaire (ESAT) [16,17] and the Checklist for Early Signs of Developmental Disorders (CESDD) [18], with M-CHAT being one of the most frequently used tools across published studies conducted in Europe.

Similar to CHAT, all of these instruments are based on a conceptual analysis of early communication development and ‘red flags’ commonly described in scientific literature as early ASD indicators, most of which stress difficulties in joint attention, social interaction, visual contact and in playing skills as important ASD risk indicators. For instance, item 12 of the M-CHAT (Does your child smile in response to your face or your smile?) and item 5 of the CESDD (The child seldom/never smiles socially, etc.) collect information about difficulties in social interaction. Furthermore, item 5 of the CHAT and M-CHAT, item 25 of the CESDD and the observation number 3 of the CHAT assess difficulties in symbolic play. Even though the similarity in the content of the items’ and wording is notable, it is important to promote empirical studies comparing the content of the items of the different instruments, in such a manner as to analyse if they are measuring the same behaviours and also if the discriminative and predictive validity of the items is similar in the different instruments. However, this question is beyond the scope of this article. 

There is a conceptual discrepancy between the different instruments and the CHAT, in that the latter does not include items related to stereotyped behaviour, which are, however, included in the other instruments. From a universal point of view, the instruments differ in terms of: number of items; number of items devoted to identifying each one of the behavioural areas of difficulty; the characteristics of the person to be informed and the type of data that must be recorded (direct observational data such as the Joint Attention-Observation Schedule (JA-OBS) and section B of the CHAT, or responses from parents or professionals).

Screening programmes use different strategies to reduce false positive and negative cases to increase the efficacy of the process. The purpose of reducing the number of false positive cases is that it would increase the specificity of the programme and lower the overall cost of the programme. Therefore, fewer resources would be necessary for conducting a smaller number of evaluations. A strategy frequently used is to have different stages in the screening process; for instance, a verification telephone interview is conducted for positive cases in the M-CHAT questionnaire. Another strategy described in the studies analysed to minimize the number of false negatives and thereby increase the sensitivity of the programme, is to combine different tools in the screening process. This is a common practice across the different selected studies (see Table 2). Moreover, one could also opt to repeat the screening at different ages as a follow-up. The latter strategy allows the programme to become a development surveillance tool and screening assumes an evolutionary approach. 

Regarding the context of these investigations, most of the studies described in this review have implemented ASD screening programmes in collaboration with the public health system of the countries where the studies were conducted, specifically in the primary care services and children surveillance programmes. In these situations, with the research team and psychiatric units or other specialized clinics work closely together to proceed with the diagnostic evaluations of the positive cases, with the exception of the investigation of [18], in which day-care centres performed the initial pre-screening (see Table 2).

Another important aspect that should be highlighted regarding the ASD screening programmes for the general population is the estimation of their scope; the number of the total population that meets the characteristics required for the study sample and the contrast with the number of subjects finally screened. That is to say, the objective is to detect the highest number of children within the range of 14–36 months who could be given an ASD diagnosis. A population’s sample size is a difficult number to record systematically and part of this difficulty is due to a combination of human factors and the type of information resources used, such as birth records, global data from national statistical services, cohort studies, patient records from clinical services, estimation of the disorder’s prevalence obtained in other studies, proportionate sample collection, etc. Hence, this is a complicated number to estimate and is not often available to research teams. 

However, all the studies reviewed addressed this issue, some in a more specific manner, reporting mostly about low participation rates (about 40%) and others describing only the scope of the total population within the collaborating institutions with the screening, without providing the actual data (see Table 2). This is the case of the study conducted in Spain, where the ASD screening programme is based on the fact that public health services reach the entire population in Spain and have a medical check-up system for healthy infants that is universal and free. This is the reason to point out that primary care services are the appropriate context for a population early screening programme.

There is considerable variability in the estimated prevalence of ASD reported in the different studies, values ranging from 5.67 [17] to 120.03 [19] ASD cases per 10,000 people (see Table 2). These data should be interpreted with caution, as the values offered in the different studies depend on the mean age during which the participants are detected and in many cases the information is not comparable because the population is selected at different developmental stages, commonly detecting fewer cases at earlier ages. For instance, the study carried out in the United Kingdom (UK) with the CHAT [14] reported an estimated 6.2 ASD cases per 10,000 at 18 months; this number is close to the 5.6 ASD cases per 10,000 estimated by the Netherlands study [17] which had a sample mean age of 15-months. Yet, in the Belgian study [18] an estimated prevalence of 60.22 ASD cases per 10,000 was reported, as all cases referred for evaluation at the end of all screening stages were taken into account. This last prevalence value is very close to the 57.9 ASD cases per 10,000 obtained in the UK using the CHAT screening tool, study that followed a two-stage implementation strategy that lasted 6 years and combined up to 5 detection and surveillance methods during the duration of the study [14]. The study by Nygren and colleagues [19] procured even higher rates, reaching 80 cases per 10,000 using a two-stage strategy and both screening tools, the M-CHAT and the JA-OBS.

An explanation for the variability in ASD prevalence rates is that not all programmes use a strategy to detect false negatives cases, owing to the great economic cost of classifying all participants correctly within the programme and also due to the low participation rate in some studies [14]. Another possible explanation is the high dropout rate from screening programmes during the programme’s different implementation stages, as some families prefer to wait and see whether their children improve before continuing with further evaluations, or because at the time of screening parents state no concern regarding their child’s development [18]. In any case, the results concerning prevalence from the different programmes suggest that those that follow a multi-stage strategy, which extends over time, achieve higher rates, as they cover a wider age range. This conclusion is consistent with the results available in scientific literature on the age of onset variability and in the different evolutionary trajectory of the children who come to have autism. Even though studies about the evolutionary trajectories of early autism symptoms onset are generally based on results obtained from studies with high-risk populations (i.e., siblings of children with autism) [22,23,24], these results suggest that symptoms can manifest in different developmental stages during the first years of life and with different severity levels. This indicates the need for screening programmes to assume an evolutionary perspective, combining procedures and instruments that allow detection from very early ages until the end of early childhood.

The most commonly used parameters in the appraisal of ASD screening programmes are sensitivity (Sen) and specificity (Spe), positive predictive value (PPV) and negative predictive value (NPV). Sen and Spe are intrinsic values to detection tests and have less dependence on prevalence than PPV and NPV. The values of Sen and Spe measure the diagnostic discrimination of a screening tool in relation to “gold standard” criteria, which allows the effectiveness of different tools to be compared and also verify that a screening tool has similar results when applied in different countries, or regions, if administered in a standardized manner. Almost all selected studies provide Sen and Spe parameters. The greatest limitation of these parameters is their lack of usefulness in clinical practice, since with this only information the probability of a person actually having ASD cannot be known. On the other hand, PPV or NPV measures report the probability of an individual having or not having the disorder based on whether they have tested positive or negative. The PPV addresses the likelihood that an individual with a positive result has the disorder. In contrast, NPV indicates the probability that an individual with a negative result does not actually have ASD. As shown in Table 3, studies using CHAT and M-CHAT point out that the probability of an individual having ASD, if given a positive test result, may be between 19% and 60% (PPV between 0.19 in [20] and 0.59 in [14]). Likewise, if the result in one of these tests is negative, the probability that they do not have ASD is 99%.

The data provided by PPV and NPV are more clinically useful than the values of Sen and Spe; however, these measures are directly related to the disorder’s prevalence rates obtained in the study. Thus, if the prevalence of the disorder in the population is low, PPV will be low, despite how sensitive and specific the screening test is, as is the case in the Spanish study [20]. Therefore, in population screenings in which the prevalence of the disorder is low, many individuals with a positive test result happen to be false positives. In contrast, if the prevalence of the disorder is high, the NPV is low, because detecting fewer people with the disorder will increase the number of false negatives.

As prevalence determines PPV and NPV values, these cannot be used as indexes to compare two different screening programmes, or to extrapolate results from one study from another. Thus, it is necessary to use other indexes of evaluation that, besides being useful in clinical practice, do not depend on the prevalence in the studied population. The parameter proposed by the scientific literature is that of likelihood ratio (LR). This concept measures the probability of a concrete outcome (positive or negative) according to the presence or absence of the disorder and depends on the test’s sensitivity and specificity, hence its usefulness when comparing screening tests. Only two of the selected programmes in this study provide LR data (see Table 3). Therefore, an effective comparison of the different programmes in relation to their psychometric properties is not possible.

According to the data collected and the proposed considerations about the psychometric properties of the screening tools, it can be suggested that almost all studies show reasonable specificity and sensitivity values; however further follow-up research should be performed to confirm that cases that are discarded by these procedures are in fact no ASD cases and that ASD positive cases have been also correctly identified by the different screening programmes. 

In the future, progress in screening tools, especially in order to be able to assess their relative effectiveness and make more befitting decisions about the feasibility of their usefulness in clinical practice, requires that studies provide psychometric data with values that are independent of the prevalence rate procured in the studies themselves, using parameters as likelihood ratio. And since these values are estimated for a specific sample, obtained in a specific place and time interval, it is recommended for these values to be accompanied by confidence intervals, as in study [18], to facilitate a better interpretation, taking into account that smaller samples will estimate less accurate values [25].

The follow-up of the detected cases in the screening programmes is an aspect that almost all the reviewed studies identify as being of great help to confirm doubtful diagnoses at early ages, where ASD symptomatology can be confused with other developmental disorders. Furthermore, re-screening at different ages helps in the detection of false negatives (see Table 2). However, it is rare to find screening studies that report data on children’s communicative and social skills, intelligence quotient (IQ), or language level at follow-up. Of all the analysed studies only the study conducted in the UK [14] provides such data, yet, it does not specify whether the evaluated subjects had received any type of intervention during the time prior to follow-up. This information would be essential for providing evidence that ASD early detection bolsters intervention at earlier ages and therefore, this may result in an improvement of the ASD symptomatology.

A poorly researched issue in the described studies is the ethnic, socio-economic and cultural characteristics of participants, as well as the service accessibility, in the sample population where the screening programmes were carried out. Each particular case has different possibilities of being detected in a different screening stage, receiving an ASD diagnosis or not, responding to a treatment based on factors that are not random, such as services accessibility, the medical-patient relationship, socio-economic status of the families, their place of residence, etc. [26]. Although many studies do not offer any data with respect to this, other characteristics have been found among diagnosed ASD cases, such as that fact that parents (especially mothers) have a high level of education [17,18,21]. In the Swedish study [19], an interesting fact about the role of immigration was reported, stating that in 50% of ASD cases both parents were not descendants of people born in Sweden. Information regarding these issues could help to better understand the risk factors and even the aetiology of ASD. In addition, it should also be examined if families who have access to better services are somehow influenced by: (a) whether they reside in more developed and/or thriving communities; (b) whether they have higher purchasing power; or (c) their educational/cultural level. For this reason, universal screening has a very important role in reducing healthcare services access inequality. Children with ASD who come from low-income families, or who live in rural areas, for example, have a significantly higher risk of receiving a late diagnosis, or even worse, the risk of never receiving a formal diagnosis [27,28]. Thus, in addition to actions directed at increasing ASD awareness and the proper training of parents and health professionals, universal screening for ASD can be very useful for reducing social inequality in the identification and treatment of children with ASD.

## 3. Strengths and Limitations of European Population-Based ASD Screening Studies

Most of the studies seem to agree on the fact that ASD universal screening helps to identify children with this disorder at an early age and several studies point out that the majority of false positive cases are children who have development problems (see Table A1 in Appendix A). Therefore, autism population screening programmes would also assist in detecting other neurodevelopment problems and reduce the age of diagnosis in many non-ASD cases as well. The early detection of neurodevelopmental disorders may result in a faster access to a wider range of resources and services, such as early intervention, which could have a significant impact in improving communicative and social abilities, ultimately reducing symptomatology, a fact that has been demonstrated in several studies [29,30,31]. A limitation regarding this matter has been pointed out by the USPSTF working group [32], which highlights that ASD early intervention studies mainly recruit children from ASD specialized services and not from universal screening programmes. Therefore, they argue that the results from these treatment efficacy studies “may not be generalizable” to children identified by universal screening programmes, which they describe as “younger and possibly less affected children.” Thus, there is insufficient evidence as there are very few high-quality studies (randomized control trials) on the efficacy of early intervention programmes with children from universal screening programmes, which is understandable to a certain extent because it is rather difficult to ethically justify which true positive case should be included in a waiting list to receive treatment, after the child and his/her family have already gone through the early detection programme. Regarding the lack of studies with younger children and children with less severe symptomatology, it may be inferred that children with less symptomatology should respond better to the intervention. In addition, there is no evidence to support that children identified through universal screening, rather than based on parents’ concerns, have a less severe affectation.

However, the lack of studies that provide evidence on early intervention’s effectiveness for children detected through screening programmes is part of the big question of how to demonstrate the value of the complex clinical process of ASD detection, diagnosis and intervention, according to [26]. These authors report that ASD is very difficult to manage within the public health systems, due to the great variability in the initial manifestations of symptoms (both in terms of initial onset and intensity of symptoms). In addition, the diversity of developmental patterns can influence the stability of the diagnosis. This would require more complex interventions and consequently, these interventions should be adjusted to the individual’s needs in terms of content, application methods and professionals involved [33]. Nonetheless, it should be noted that all the described studies have been conducted in collaboration with public health systems, early intervention programmes and even day-care centres (see Table 2), which indicates that such coordination is possible. Some of them underline the importance of raising ASD awareness and highlight the relevance of adequate training in detection programmes, both for professionals involved in their application and for society in general [19,20]. Furthermore, ASD screening programmes have several stages at different ages and most of these practices are framed within development surveillance programmes. All these measures seek to identify the largest possible number of ASD cases within the studied population and to reduce false negatives cases (see Table 2 and Table A3 in Appendix C). There are some screening tools, such as the M-CHAT, that even have a verification procedure to minimize the number of false positives, which reduces costs as less diagnostic evaluations are performed.

Another important limitation that must be noted is related to the design of prospective observational studies in general population cohorts. Data collection is always affected by participation rates of both professionals who apply the screening tools and the individuals who agree to participate in the study. Furthermore, studies are also affected by participants’ dropout rates during screening stages (due to a number of different reasons) as well as sample selection bias. These variables are very difficult to control, having as a result, estimated prevalence values that should be interpreted with caution, as well as the psychometric measures of the programme’s effectiveness (see Table A3 in Appendix C). 

## 4. Early Intervention Treatment Programmes

As discussed throughout this review, it is important to measure the effectiveness of ASD early detection tools and programmes, in order to estimate the number of diagnosed cases and assess the available resources destined for early intervention should aim. In light of the need to respond to the high ASD prevalence with effective treatments, over the last decades scientists have worked diligently to develop efficient early intervention programmes for young children with autism and even though there are a well-known number of approaches to treat ASD, no approach has been identified to improve the functioning of all treated children with ASD [34]. However, there is a large body of research dedicated to evaluating the different types of evidence-based early intervention practices used to treat autism. For instance, the findings of a study conducted by Wong and colleagues [35] suggest that there are two broad classes of intervention approaches, classifying them in comprehensive treatment models (CTMs) and focused intervention practices (FIPs). Comprehensive programmes are a set of practices designed to achieve a wide-ranging learning or developmental impact on the core deficits of ASD, while focused interventions are designed to address a single skill or goal. Furthermore, another study on early intervention approaches points out that FIPs usually happen over a moderately short period of time and such programmes comprise reinforcement, peer-mediated intervention and prompting. In contrast, CTMs occur over a prolonged period of time, often a year or longer and are characterized by the intensity of their application [36]. Also, it is important to note that regardless of their method these early intervention practices for ASD integrate both behavioural and developmental methods, focusing on the development of social and cognitive skills, such as communication, shared attention, attention and regulation, imitation and symbolic play [37]. 

**Figure 2:**
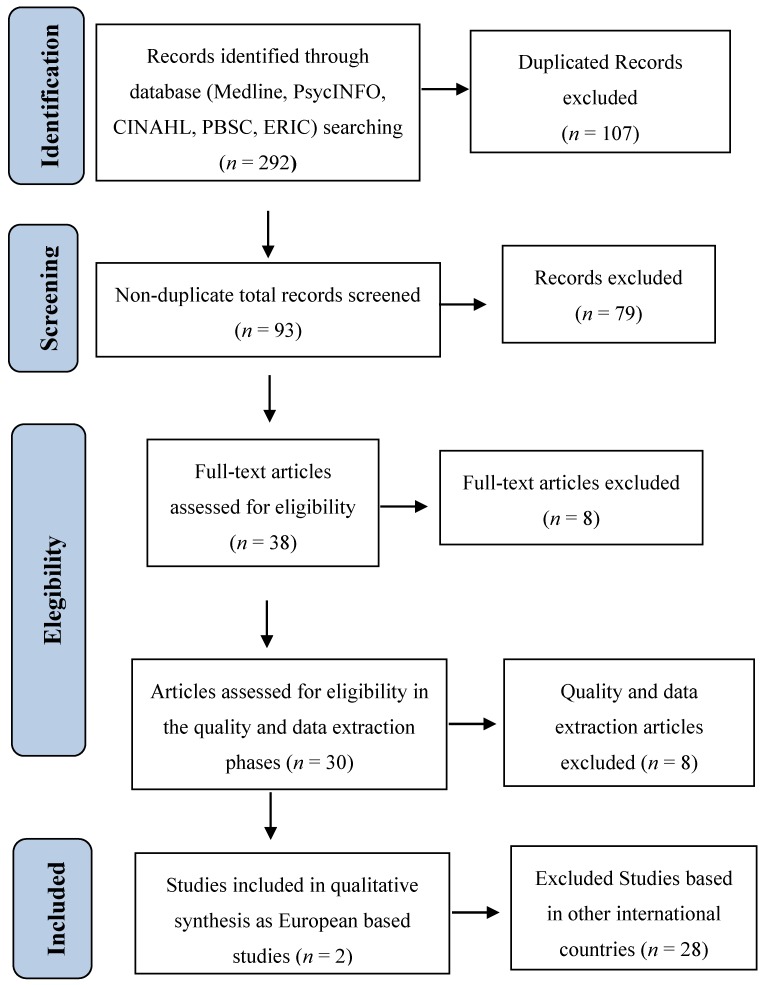
PRISMA flowchart representing the identification and selection of studies.

Another important aspect of CTMs is that they typically operate following multidisciplinary approaches, integrating principles of applied behavioural analysis (ABA) and/or discrete trial training, developmental and relationship-based procedures, among others; yet in spite of the increased use of ABA over the last decades, misrepresentations and misunderstandings concerning this approach continue to be a topic of debate amongst scientists [38]. Although there are differing scientific opinions with regard to the effectiveness of ABA techniques, in a review of the trends and topics of early intensive behavioural interventions (EIBIs), a type of CTM that is widely ruled by ABA [39] it has been proposed that ABA could possibly be the most effective treatment approach for autism. As shown in Table 4, a summary of the data obtained from a systematic literature review carried out within WP2 in the framework of the ASDEU project [9], the retrieved studies identified and evaluated the efficacy of well-established CTMs and provided information regarding intervention outcomes on the core deficits of autism (see Figure 2 for the study selection process and Table A2 in Appendix B for the electronic search strategy; more details of the methodology protocol can be provided upon request).

The systematic evaluation of CTMs studies indicates that there is scarce scientific research evaluating European programmes, as 12 of the 14 studies found in this review were designed and implemented in America (United States of America and Canada). Also, only two studies were conducted in Europe (Italy and United Kingdom), published in scientific English peer-review journals (to assure the replicability of the study), of which only one evaluated a European programme, The Scottish Centre for Autism Preschool Treatment Programme (SCA) [9,45]. Nonetheless, these findings shed light on the most commonly used treatment programmes, both in Europe and in other non-European regions. According to the literature, in some European countries programmes such as Treatment and Education of Autistic and Related Communication Handicapped Children (TEACCH), Early Start Denver Model (ESDM), Parent Delivery of the Early Start Denver Model (P-ESDM), Early Intensive Behavioural Intervention (EIBI), Early Behavioural Intervention (EBI), Life skills and Education for Students with Autism and other Pervasive Behavioural Challenges (LEAP) have been implemented and studied to measure their effectiveness within the European context. It is important to note that most of the abovementioned programmes, though typically funded by public grants, are carried out in academic institutions, making it difficult to infer whether they could be effectively implemented in the public health sector. TEACCH is a comprehensive programme for children with autism that was established at the University of North Carolina in 1966; its creators define the treatment method as a collaborative global approach where parents and professionals work together [47,48]. A standard TEACCH intervention consists of an initial assessment of the individuals’ abilities through standardized tests, where the results of such an evaluation provide the foundation for the development of a curriculum that is consistent and suitable for the individual needs of each child [48]. The programme’s intensity, that is to say, the weekly number of hours of treatment given, can be classified as low, low-to-medium and medium intensity and the method also includes a natural environment (home) intervention. Further, this at-home method may reduce parental stress levels as well as the parents’ perception of their child’s maladaptive behaviours [40]. This method has also been studied in school-based settings and the results indicate that children in the TEACCH programme benefit from this approach, as students made gains across ASD domains and/or reductions in autism characteristics [41,49,50]. These findings shed some light on the feasibility of implementing the program in larger, community-based settings; yet, a limitation often noted in most studies is the impossibility of randomly assigned participants to the intervention programmes. 

Another model that has been examined in this review was the Early Intensive Behaviour Intervention (EIBI), as there are a number of studies that sustain that this intervention, which works on the principles of applied behaviour analysis (ABA), is effective in treating the core deficits of ASD [51]. However, when implemented in community-based settings EIBI yields mixed results in terms of its effectiveness [52]. Nevertheless, empirical research has also shown that when implemented for a prolonged period of time, EIBI can have robust results on measures of intelligence, expressive and receptive language, daily living skills and positive behaviour [51]. Similar to other well-known EIBI models, the Nova Scotia Early Intensive Behaviour Intervention (NS-EIBI) is a programme that was created for young children with autism to explicitly address the challenge of providing feasible, sustainable evidence-based intervention that would target the core deficits of autism in the socialization and communication domains [42]. This intervention combines parent training and naturalistic one-to-one behaviour intervention using pivotal response treatment techniques and results from a study aimed at identifying the effectiveness of the programme showed that behavioural problems decreased significantly over 1-year of treatment and that children also showed improvement in multiple developmental domains, such as language, communication and adaptive behaviour [42]. 

However, even though both TEACCH and EIBI are well-known models, amongst the widely used programmes we can also find the Early-Start Denver Model (ESDM) a comprehensive early behavioural intervention for toddlers and preschool-aged children developed by psychologists Sally Rogers, Ph.D. and Geraldine Dawson, Ph.D. The model combines applied behaviour analysis principles with developmental and relationship-based approaches and was created to address the needs of young children with ASD as young as 12 months of age; where treatment takes place in a familiar environment (home) and intervention sessions are delivered by trained professionals and parents [46]. Empirical studies evaluating the effectiveness of the ESDM and the ESDM Parent Delivery version indicate that this model is characterized by a rigorous, structured curriculum that is followed and implemented according to the programme’s guidelines and research findings suggest positive treatment outcomes as children in these high intensity interventions have shown improvement in cognitive abilities and domains of socialization, language, daily living skills and motor skills IQ and adaptive behaviours [43,46,53]. Nonetheless, even though these programmes provide evidence of positive clinical outcomes on the core deficits of ASD, it does not seem to improve Autism Diagnostic Observation Schedule (ADOS) scores [46,54]. In regard to this model it is important to note that even though it widely used in America, there is little information regarding whether it could be successfully implemented within the European context. 

## 5. European Early Intervention Comprehensive Treatments

Considering the amount of data pertaining to the investigation of CTMs effectiveness in America, it is somewhat daunting, though perhaps not that surprising, that in European countries there remains the need to conduct more empirical studies measuring the usefulness and implementation feasibility of these interventions within the European healthcare system. Nonetheless, in some European countries, such as Italy and the United Kingdom, studies measuring the efficiency of already established programmes such as TEACCH, LEAP and EIBI have been conducted [45,55], indicating that these models have positive intervention outcomes as they are characterized by well-structured curriculums designed by experienced professionals who take into account each child’s specific needs, designing and implementing the intervention protocol accordingly. In fact, across studies the evaluation of these models pointed out that most commonly used comprehensive programmes operate on Discrete Trial Training principles, such as Applied Behavioural Analysis (ABA) and Pivotal Response Treatment (PRT); Picture Exchange Program System (PECS), which supports the notion that behavioural interventions do seem to provide positive results in ameliorating the clinical symptomatology of children with ASD, as well as the social and communication impairments that come with it. Moreover, most studies evaluating ASD treatment effectiveness highlight the importance of parent involvement, either as at-home therapists or onsite co-therapists who would deliver part of the intervention and the role it has in both the children’s improvement and in reducing parents’ ASD-related stress levels. Furthermore, these studies also suggest that those programmes where parents have an active role, either as lead therapists or as co-therapists, seem to have a greater effect size than those in which there is only a therapist and/or in which the intervention is carried out by a teacher in a school setting with a therapist’s assistance [9]. Yet, in intervention practices, such as the TEACCH and LEAP programmes, teacher involvement does seem to play a key role in positive intervention outcomes, as well as teacher training, which varies across studies (between 10 and 20 h of training). The implementation of in-class intervention also shows positive results, as well as training of typically developing children in comprehensive social skills that would facilitate the social and communicative behaviours of their peers with ASD [40,41]. 

Although high-intensity comprehensive treatments have been associated with positive intervention results, procuring studies that investigate the effectiveness of such programmes in reducing ASD symptoms and improving the core deficits associated with the disorder is more common than finding scientific work studying low-intensity programs. However, just as comprehensive treatments have positive intervention results, so do focused intervention practices, as suggested in [56], which evaluate the social-communicative effects of the Picture Exchange Communication System (PECS) in treating children with ASD. In this study children were assessed pre- and post-intervention and the findings indicate that even though there were no significant group differences in standardized tests scores at baseline (T1), post treatment outcomes (T2) show significant improvement on the VABS social-domain and social communicative abilities [47]. Moreover, an ongoing study investigating the efficacy of focused interventions [9] found a positive correlation between the time of treatment and intervention effectiveness, which supports the notion that the higher the intensity of the intervention the better outcomes achieved. 

Although the replication and implementation of comprehensive and focused programmes in countries of the European Union has shown positive results [40,57], according to a literature review conducted under the ASDEU project [9] not many studies have proposed the development and implementation of a European comprehensive and/or focused programme, except for The Scottish Centre for Autism Preschool Treatment Programme (SCA). This treatment curriculum, run by a tertiary service within the Department of Child and Family Psychiatry at Yorkhill Hospital, Scotland, has a social-developmental approach and follows a well-structured curriculum, as it is supervised by the programme’s creators, its daily organization is arranged by a programme coordinator and is integrated by a multidisciplinary team of therapists (nursery nurses, teachers, occupational therapists, speech and language therapists) all well-trained in the programme’s therapeutic approach [55]. Moreover, a study conducted to examine the effectiveness of the treatment suggested that having access to this intervention resulted in positive outcomes for children with ASD, since children receiving the intervention significantly improved in the measures of social interaction, imitation, daily living skills, joint attention and adaptive behaviours [45]. 

Yet, with regard to the subject of ASD early intervention, two aspects must be carefully evaluated: (1) the profile of the professionals and their training in ASD; and (2) the perspective of family members regarding the intervention, such as access to treatment, how the intervention is administered, its duration and post intervention results. In light of the poor data on these two aspects, work package 2 of the ASDEU project [9] launched two surveys within the European union context, one for families and one for professionals, for better understanding this matter. The data collected from these surveys indicates that the geographical location of the intervention centres is one of the most important elements to be taken into account to reduce treatment delay, as the time it takes to get to these centres is often one of the main challenges families face when accessing treatment services [9]. Furthermore, another limitation stressed by participants is the facilities where early interventions take place. Those responding to the surveys also emphasized the importance of better informing the families about the disorder and also pointed out how relevant it is for professionals working directly with individuals with ASD to have proper training. Additionally, these findings indicate the common view that there should be a shared effort to bring ASD awareness and to promote early intervention coordination, proposing intervention guidelines for different age groups and establish and well-defined procedure. Lastly, the data collected from [9] emphasizes the importance of providing evidence-based intervention methods, which is a subject that demands further research.

## 6. Conclusions

With regard to early detection, the review of screening procedures and experiences in Europe seems to suggest, as a main concern, the feasibility of standardized and systematic actions to improve the early identification of children with ASD, reducing the age of diagnosis. Thus, in the programmes analysed in the present study, detection begins very early, reaching diagnostic ages at an age close to 24 months. However, in Europe, the age of ASD diagnosis is on average at age 3.5, with great variability between countries [58], an age which remains to be quite late. The age of diagnosis has certainly been reduced in the last 25 years but the experiences analysed indicate that it is should be possible to further reduce this delay [19,20]. The causes of the delay can be very diverse and do not necessarily have to be associated with a lower development of diagnostic services [59]. Some sociodemographic characteristics also have an influence, such as the place of residence of the child, the socioeconomic level of the family, or structural limitations of the services that give rise to long waits for the child to be diagnosed [28]. The aforementioned causes are variables that intervene in the temporary space that exists between the parents’ first concerns (if these take place) and the time of diagnosis. Some key milestones during that period could be the moment when the first concerns of the parents arise, as well as the first contact with the paediatrician, the assessment of the symptoms by the paediatrician, the referral to the hospital, the first visit to the hospital and the diagnosis and subsequent referral to the treatment program. It is probably necessary to deepen the understanding of the delay in identification and diagnosis but the causes identified so far can be addressed in a simple manner through specific actions to improve detection adjusted to each country or region, as is the case of the screening studies reviewed in this article. Efforts to reduce the delay should take into account the causes that are known and should include, among others, actions aimed at rationalizing the process from the first concern of the family to the final diagnosis, modify community resources and state policies through of laws or guidelines of good practice and to promote the education of parents and professionals on the early recognition of development problems. All this can be done to a large extent with early detection programs, such as those reviewed here, which are clear examples to follow. Although it is still necessary to adapt the most convenient actions according to the characteristics and needs of each country.

Secondly, findings from this review indicate that the screening programmes that yield better results combine procedures and instruments that allow detection from very early ages until the end of early childhood. This multi-stage approach in early detection is useful because it improves the inherent problem pertaining to age of symptoms onset and symptoms’ intensity, as they may vary from case to case. The strategy of combining different identification procedures during different ages in a single screening programme deserves to be a research objective, because its practical application could also serve as a more effective way to reduce the number of false-positive and false-negative cases within the framework of a development surveillance strategy. However, a development surveillance strategy without prior proper training in detection tools can invalidate the psychometric properties of these tools [60]. Therefore, it is necessary that the research and practical application of the screening programs include the goal of improving the training of healthcare professionals, increase the support of private practices and also that health services authorities assume detection as a general responsibility by promoting the creation of comprehensive networks. Training aimed at professionals should not only help improve knowledge about the use of specific detection tools but it should also provide professionals with resources to adequately recognise the signs of concern. This training should be combined with systematic ASD awareness campaigns aimed at families describing ASD warning signs and the advantages of early identification. This complementary strategy could be very important as it could provide a platform to help families express their concerns regarding the disorder with confidence. Moreover, the findings from the review suggest that the inclusion of a case follow-up system, not only aimed at detecting false negative cases, would also allow professionals and parents to identify signs of symptoms improvement due to early intervention. Hence, this strategy could help both professionals and families to have a more positive perception of autism early detection and also improve the knowledge on the development of the symptoms of autism and the study of ASD profiles (cognitive, communicative, etc.).

Thirdly, the reviewed studies show different examples of how to conduct a systematic identification of children with ASD; however, they are hardly comparable to each other as programmes do not use the same parameters to appraise screening tools and procedures. Researchers and professionals who assess detection tools and methods should provide data that report on its psychometric properties, which would allow to efficiently evaluate the relative effectiveness of each procedure and to compare different programmes using the same parameters. Thus, studies aimed at improving detection should provide values of Sen and Spe and VPP and VPN, which may allow professionals to know the probability of an individual having ASD or not depending on the results obtained on the tests. Given that the values of VPP and VPN depend on prevalence, it is very useful that detection studies also provide values of likelihood ratio, a parameter that measures the probability of a result (positive or negative) according to the presence or absence of the disorder, which does not depend on prevalence but on the sensitivity and specificity of the test and hence, its usefulness when comparing screening tests. Providing these psychometric values could be very useful in when making political and administrative decisions regarding what type of tool or programme could be better to promote in the European context.

With regard to early intervention programmes, so far there is no specific European model, since the models found in the scientific literature describe and analyse the results of the application of programs developed in other regions of the world. However, this seems to indicate that the European scientific community is following the example of those studies that have shown to be effective. Furthermore, it can also be inferred that most professionals in Europe take these experiences into account; although it is still necessary to demonstrate through the publication of scientific reports that in Europe the early intervention of ASD is comparable to that of the rest of the world. Nonetheless, the literature devoted to the description and evaluation of early interventions for autism has become quite vast in recent years. The various treatment models addressed in this review may allow the formulation of some recommendations that could be taken into account in the field of ASD early intervention. First, there seems to be an increasing convergence between behavioural and developmental methods (i.e., ESDM) and research findings suggest that the focus of early interventions for ASD is directed toward the development of skills considered “pivotal” such as shared attention, imitation, communication, symbolic play, cognitive abilities, attention and regulation. Second, the literature proposes some guidelines for effective treatments such as: (1) starting as early as possible; (2) minimizing the gap between diagnosis and treatment; (3) providing at least 3–4 h of treatment per day; (4) centring on family involvement; (5) choosing among the behavioural/developmental approaches depending on the child’s profile and/or treatment response; (6) encouraging spontaneous communication; (7) promoting skills through play with peers; (8) finalizing the acquisition of new skills, generalization and maintenance in natural contexts and (9) supporting positive behaviours rather than just tackling challenging behaviour. Moreover, all studies examining the effectiveness of early comprehensive models urge the importance of adequate programme structuring and implementation by a lead experienced professional (psychologist, specialized interventionist) and a team of well-trained therapists (psychologists, speech therapists, graduate students, teachers). In this sense, it is likely that all efforts aimed at improving the training of European professionals could have very favourable consequences for young children with ASD receiving intervention. It is important to note that most comprehensive treatment programmes found in the scientific literature that have demonstrated efficacy follow a behavioural approach, such as Applied Behaviour Analysis (ABA) or Pivotal Response Training (PRT) and in some instances, these models could also take on a more naturalistic approach to target core ASD deficits. Moreover, from the review we can infer that when evaluating treatment effectiveness, studies must comply with certain features such as the presence of a comparable control group, the presence of precise diagnostic criteria, periodic evaluations during treatment and evaluation of different developmental aspects (severity of autism, cognitive and language development, global functioning and quality of family life and parent-child interaction). Furthermore, the presence of a specialized team in the management and treatment of autism and the active collaboration of the family in treatment planning and execution could also be considered as two pillars of any treatment program [61]. Even though for the purpose of this review only early intervention comprehensive treatment programs were examined, the role of focused practices must also be acknowledged. Focused interventions, including those programmes aimed at improving parents’ ability to develop communicative and social skills, also aim to improve the problem behaviours of children with ASD and reduce the severity of symptoms, such as the program developed by [62,63]. These promising intervention practices are the subject of analysis within the framework of the study on which this article is based and the results of this analysis will be presented in an article currently under preparation. However, several studies suggest [64,65] that conducting studies that will provide empirical evidence to appraise treatment characteristics, such as timing, intensity, intervention delivery and duration remains to be a research priority. 

There is general agreement in the autism field to find the best detection, diagnostic and intervention tools and programmes. However, political authorities, parent organizations, professionals and researchers alike also have a responsibility to support, promote and conduct controlled studies that can later be transferred from these controlled contexts to clinical practice, within the framework of protocols protected by public policies that acknowledge the needs of the families of children with ASD and that provide the public services with the required resources to coordinate and evaluate the efforts for early ASD identification and intervention, for the sake of young children with ASD.

Finally, it must be highlighted that the search conducted for this review has mainly focused in European studies and thus, it is important to note and acknowledge that there is a copious amount of international research on screening and early intervention programme, which could without a doubt be useful within the European context. However, examining this concept was out of the scope of this study. Further, more research should be devoted to the investigation and comparison of screening and early interventions practices from other international backgrounds with the objective of testing feasibility and exploring other good practices to be applied not only in European contexts but anywhere else.

## Figures and Tables

**Table 1:** Screening tools characteristics used in European studies for early detection of ASD.

Screening Tools	Developmental Areas Measured	*N* Items	Application		
			Age (Months)	Time (Min)	Admin Procedure
Checklist for Autism in Toddlers [13]	Pretend play; Proto-declarative pointing; Joint-attention; Social interest and Social play.	9 + 5 ^1^	18	5–10	Rated by parents and health practitioners
Modified-Checklist for Autism in Toddlers [15]	Sensory abnormalities; Motor abnormalities; Social interchange; Early joint-attention/Theory of mind; Early language and communication	23	16–30	5–10	Rated by parents
Early Screening Autistic Traits Questionnaire, [16,17]	Pretend play; Joint-attention; Interest in others; Eye contact; Verbal and non-verbal communication; Stereotypes; Preoccupations; Reaction to sensory stimuli; Emotional reaction and Social interaction	14	14–15	10	Rated by parents and a child care worker
Checklist for Early Signs of Developmental Disorders, [18]	Different target behaviours but no specific developmental areas	25 + 4 ^2^	3–36	-	Rated by child care workers
Joint Attention-Observation Schedule, [19]	Joint-attention	5	20–48	5–10	Nurse observation schedule

ASD: Autism Spectrum Disorders; Min: Minutes; ^1^ Nine items rated by parents and five observational rated by health providers; ^2^ 25 items related to ASD target behaviours and four items related to language.

**Table 2:** European ASD screening studies characteristics.

Screening Studies & Location	Setting	Estimating the Scope of the Screening	Estimated Prevalence for ASD	*N* Total *	Gender F (M)	Age Mean (SD)	Follow-Up	Social, Cultural & Economic Factors
Baird at al., 2000 [14] CHAT South East Thames Health Region, England. UK	Primary health providers (1-stage) screened a birth cohort of children, during a routine 18-months developmental check or the CHAT was mailed to parents. Positive screen cases were re-screened by the research team (2-stage) and if positive they conducted further assessments	Yes. Of the total population of 40,818 children aged 18-months, a 39.8% were screened	57.9 ASD cases per 10,000 people ^1^	16,235	N/R	18.7 (1.1)	Yes. Conducted throughout the following 6 years	N/R
Dietz et al., 2006 [17] ESAT Province of Utrecht, mostly urban. The Netherlands	Primary care system for surveillance of developmental problems. The well-baby clinics applied a pre-screening test. (Attendance to these clinics is not compulsory). If the children screened positive, a home visit screening will be performed by a trained psychologist, if this is positive the family will be invited to the Department of Child Psychiatry for further assessments	Based on the estimated ASD prevalence in an initial study [9], the overall design size was to screen 30,000. A total of 31,724 children were screened (106%)	5.67 ASD cases per 10,000 people	31,724	37% (63%)	14.91 (1.37)	Yes. Re-screen at 24- and 42-month of the negative cases and re-evaluation of the positives at the same age	The population is mostly well-educated, only 13% of the population has just primary education.
Dereu et al., 2010 [18] CESDD, ESAT, SCQ & FYI Flanders. Belgium	70 day-care centres screen children older than 3 months with the CESDD for developmental problems, if positive further screening for ASD using parent reports was performed. Positive cases in these reports were referred to Developmental assessment at the University Lab. Based on the clinical judgement of the research group positive cases were referred to specialized university based autism clinics or diagnostic centres for developmental disorders.	Based on the children attending day-care centres in Flanders, a 34.48% were screened	60.22 ASD cases per 10,000 people	6808	48% (52%)	16.7 (8.19)	No but the authors highlight that further follow-up of the total sample could help to find missed cases	Limited demographic information was gathered. The educational level of the mothers slightly high.
Canal-Bedia et al., 2011 [20] M-CHAT Salamanca and Zamora and Madrid Health Area No. 1 Spain ^2^	Screening programme within the Spanish National Health System (SNHS) attended children aged 18–36 months who were coming to the mandatory vaccination program and /or well-baby check-up examination. The research team perform the M-CHAT phone interview of positive cases in the questionnaire and further assessments for diagnosis. Cross-sectional study design	N/R. Though the authors described that the SNHS covers 100% of the population, regardless of their level of income or employment status.	29.19 ASD cases per 10,000 people	2055	46% (54%)	Age range 16–30 months	No but authors highlight the importance of follow-up studies to ensure cases classification	N/R
Nygren et al., 2012 [19] M-CHAT & JOBS Gothenburg, a metropolitan area Sweden	Screening procedure within the existing developmental surveillance programme at the Child Health Centres. Diagnostic procedure conducted by the neuropsychiatric specialist clinic	Yes. The total population of 2.5-year-old children in 2010 was estimated at 6220, of which 80% were screened. However, only 3.999 families participated in the study	120.03 ASD cases per 10,000 people	3999	48% (52%)	29 (0.5)	N/R	The 50% of ASD cases both parents were of non-Swedish descent
Stenberg et al., 2014 [21] M-CHAT Norway	Norwegian Mother and Child Cohort Study (MoBa) that is a prospective population-based pregnancy cohort established by the Norwegian Institute of Public Health; the Autism Birth Cohort (ABC) study (nested case-cohort designed to identify cases of ASD in the MoBa); and the Norwegian Patient Registry (NPR).	Yes. Enrolment in the MoBa from 1999 to 2008 got around 109,000 children. The participation rate was about 38.5%, however 73% of MoBa participants completed the 18-months screening questionnaire	33.25 ASD cases per 10,000 people	52,026	49% (51%)	18	Yes. Children data collection was performed from birth until 9 years and 4 months.	Authors reported some characteristics like: Maternal education years
Baduel et al., 2017 [10] M-CHAT & CHAT Midi-Pyrénées area. France	Implemented within the French health-care system during the 24 months well-child visit at their paediatrician’s office or at the day care centres. Evaluations took place either at the laboratory or at the child’s day-care centre.	N/R. Authors informed about a low rate of participation, only 14 paediatricians from the 175 and 62 day-care centre staff from 400. However, only the 16.5% of children under 3 years benefit from day-care centre services	68.17 ASD cases per 10,000 people	1250	47% (53%)	24	Yes. At 30 and 36 months with CHAT observations items	N/R

F: Female; M: Male; SD: Standard deviation; N/R: Data not reported; ^1^ Prevalence rate calculated after follow-up; ^2^ Data from the stage 2-general population; * N Total before exclusions due to incomplete procedures or others.

**Table 3:** Psychometrical properties of European ASD Screening studies.

Ref.	FN	FP	TP	TN	Sen (95% CI)	Spe (95% CI)	PPV (95% CI)	NPV (95% CI)	LR+ (95% CI)	LR- (95% CI)
Baird at al., 2000 [14]	74	14	20	16,127	0.21 (0.14–0.31)	0.99 (0.99–0.99)	0.59 (0.41–0.75)	0.99 (0.99–0.99)	*	*
Dietz et al., 2006 [17]	*	55	18	*	*	*	0.25 (0.16–0.36)	*	*	*
Dereu et al., 2010 ^1^ [18]	8	419	33	6348	0.80 (0.65–0.91)	0.94 (0.93–0.94)	0.07 (0.05–0.10)	0.99 (0.99–0.99)	12.98 (10.89–15.52)	0.21 (0.11–0.39)
Canal-Bedia et al., 2011 ^2^ [20]	0	25	6	2024	1 (0.52–1)	0.98 (0.98–0.99)	0.19 (0.05–0.33)	1 (0.99–1)	*	*
Nygren et al., 2012 ^3^ [19]	2	5	43	*	0.96 (0.84–0.99)	*	0.89 (0.77–0.96)	*	*	*
Stenberg et al., 2014 [21]	114	3804	59	48,049	0.34 (0.27–0.42)	0.93 (0.92–0.93)	0.02 (0.01–0.02)	*	4.6 (3.77–5.73)	*
Baduel et al., 2017 ^4^ [10]	6	8	12	1227	0.67 (0.41–0.86)	0.99 (0.99–0.99)	0.60 (0.36–0.80)	0.99 (0.99–0.99)	*	*

Ref.: References to the studies; FN: False negatives cases; FP: False positive cases; TP: True positive cases; TN: True negative cases; Sen: Sensitivity; Spe: Specificity; PPV: Positive predictive value; NPV: Negative predictive value; LR+: Likelihood ratio for a positive test; LR-: Likelihood ratio for a negative test; CI: Confidence Intervals; ^1^ Data estimated with a cut-of score of two signs of ASD in the CESDD; ^2^ Data from the stage 2-general population; ^3^ Data of M-CHAT and JA-OBS combined; ^4^ Psychometric properties of the combined scoring methods; * Not reported data.

**Table 4:** Summary of Studies on Early Intervention Comprehensive Treatments.

Study	Region	Program(s)	Approach	Setting	Intensity	Duration
Lidia D’Elia et al., 2013 [40]	Italy	TEACCH	Multidisciplinary	School & home	10–24 h a week	24 months
Brian A. Boyd et al., 2013 [41]	United Stated of America	TEACCH	Multidisciplinary	Public schools	-	1 school year
Isabel M. Smith 2010 [42]	Canada	NS-EIBI	Pivotal Response Treatment Behavioural	School & home	15 h a week	12 months
Sally Rogers et al., 2012 [43]	United Stated of America	P-ESDM	Denver Model Pivotal Response Treatment Applied Behavioural Analysis	University clinic	1 h a week	3 months
Annette Estes et al., 2014 [44]	United Stated of America	P-ESDM	Early Denver Model Pivotal Response Treatment Applied Behavioural Analysis	University clinic	2.6 h a week	3 months
Jeff Salt et al., 2002 [45]	United Kingdom	SCA	Social Developmental	School	8 h, every 2 weeks	10 months
Geraldine Dawson et al., 2010 [46]	United States of America	ESDM	Early Denver Model Pivotal Response Treatment Applied Behavioural Analysis	Home & clinic	20 h a week	24 months

Treatment and Education of Autistic and Related Communication Handicapped Children (TEACCH), Nova Scotia Early Intensive Behavioural Intervention (NS-EIBI), Parent Delivery of the Early Start Denver Model (P-ESDM), Scottish Centre for Autism Preschool Treatment Programme (SCA), Early Start Denver Model (ESDM) (-) Information not reported.

## Appendix A

**Table A1:** Electronic search strategy for ASD screening programmes.

“developmental disabilities” (Mesh) OR “developmental disabilities” (Title/Abstract) OR “developmental disability” (Title/Abstract) OR “developmental disorders” (Title/Abstract) OR “developmental disorder” (Title/Abstract) OR “Developmental delay” (Title/Abstract) “autistic disorder” (Mesh Terms) OR (“autistic” (Title/Abstract) AND “disorder” (Title/Abstract)) OR “autistic disorder” (Title/Abstract) OR “autistic disorders” (Title/Abstract) OR “autism” (Title/Abstract) #1 AND #2 “autistic disorder” (MeSH Terms) OR “autistic disorder” (Title/Abstract) OR “autistic disorders” (Title/Abstract) OR “autism” (Title/Abstract) OR “child development disorders, pervasive” (MeSH Terms) OR “Pervasive developmental disorder” (Title/Abstract) OR “Pervasive developmental disorders” (Title/Abstract) OR “PDD” (Title/Abstract) OR “autistic spectrum disorder” (Title/Abstract) OR “autistic spectrum disorders” (Title/Abstract) OR “autism spectrum disorder” (Title/Abstract) OR “autism spectrum disorders” (Title/Abstract) OR “ASD” (Title/Abstract) #3 OR #4 “diagnosis” (Mesh:noexp) OR “diagnosis” (Subheading) OR “Detection” (Title/Abstract) OR “diagnosis” (Title/Abstract) OR “Early diagnosis” (Mesh:noexp) OR “Early diagnosis” (Title/Abstract) OR “Early detection” (Title/Abstract) OR “Early identification” (Title/Abstract) OR “Early intervention” (Title/Abstract) OR “Early prediction” (Title/Abstract) “screening” (Title/Abstract) OR “Early screening” (Title/Abstract) OR “Mass Screening” (Majr:NoExp) OR “Mass Screening/instrumentation” (Majr:NoExp) OR “Mass Screening/methods” (Majr:NoExp) OR “mass screening” (Title/Abstract) OR “screening tool” (Title/Abstract) OR “screening tools” (Title/Abstract) OR “screening test” (Title/Abstract) OR “screening instrument” (Title/Abstract) OR “screening instruments” (Title/Abstract) OR “checklist” (MeSH Terms) OR “Checklist” (Title/Abstract) OR “Checklists” (Title/Abstract) OR “Follow-up” (Title/Abstract) #6 AND #7 #5 AND#8 Search range: 1 January 1992–4 January 2015 Language: English Academic Journals (Peer Reviewed)

## Appendix B

**Table A2:** Electronic search strategy for ASD early interventions.

Diagnostic: Autism OR ASD OR Autism Spectrum Disorder * OR Autistic Disorder * OR Pervasive Developmental Disorder * OR PDD. Intervention: intervention OR treatment OR therapy OR program OR practice OR strategy OR early intervention OR comprehensive treatment OR comprehensive model OR integral approach. Model: SCERTS OR SCERTS model OR ABA OR early intensive behavioural intervention OR EIBI OR Denver model OR ESDM OR LEAP OR Program for preschools and parents. Population: children OR young children OR pre-schooler OR infant OR toddlers. Setting: primary health care OR primary care OR health care system OR preschool OR day-care OR child day-care centres OR primary school OR children centres. #1 AND #2 AND #3 AND #4 AND #5 Publication Date: 2000-2016 Language: English Source Type: Academic Journals #7 AND #8 AND #9

## Appendix C

**Table A3:** Strengths and limitations of European ASD screening studies for early detection in general population.

Study Reference	Strengths	Limitations
Baird at al., 2000 [14]	- The study shows that ASD can be prospectively identified at 18 months of age, which has important implications for health service resources. - The CHAT used as a 2-stage procedure has an extremely low false-positive rate and the PPV for ASD is greater than 50%. - Early identification of ASD may result in considerable benefit to children and their families in many ways (e.g., Helping to make informed decisions about having more children or having earlier intervention). - Subjects were identified via 5 different methods over the course of 6 years, leading to higher ascertainment.	- Only 40% of the eligible total population were screened at 18 months and some children with profound handicap were excluded, hence the efficiency of the screen in a total population and prevalence figures are unknown in the study. - Not all the medium-risk group at CHAT-1 received a second administration and low risk thresholds were not tested because of resource constraints. - As the second administration was performed by the research team, it is difficult to know how the screen would operate when conducted by community health practitioners - No data on the reliability of the instrument. - High rate of false negatives and low PPV with the full programme.
Dietz et al., 2006 [17]	- First prospective population study to screen for ASD in children aged 14–15 months, showing that early detection is possible. - None of the false positive cases detected were found to have a typical development. - 14-item ESAT test could help clinicians when considering referral for diagnostic evaluation (screening for children at risk of ASD, Level 2). - 14-item ESAT test was found to detect ASD in different levels of cognitive functioning.	- Validity of the screening instrument ESAT was not calculated because data on the false negative cases was not sufficient. - The pre-screening test generated a high rate of false positive cases. - The screening method could fail to detect milder variants of ASD and/or ASD children with high level of development at 14 months. - High rate of drop out at this early age, because parents did not want to cooperate. - Parents hesitated to cooperate in the study because of delays in the detection procedure between the home visit and examination at the Department of Child an Adolescent Psychiatry
Dereu et al., 2010 [18]	- First prospective screening study that incorporates child care workers’ report on signs of ASD in young children. - CESDD had a better sensitivity than other tests used in random samples. - Including children with only language delay in further stages of the screening procedure helped to detect more false negatives.	- Low compliance rate at each stage of the screening procedure due to different reasons: parents decline invitation, child-care workers did not want to unnecessarily worry the parents about the development of their child, or the child was seen at another assessment centre and the parents did not want to overwhelm their child with more assessments. - Screening with the CESDD generated a large number of false positives.
Canal-Bedia et al., 2011 [20]	- First official Spanish version of the M-CHAT to be applied in Spain. - Provides evidence that ASD cases could be detected by the SNHS at around 24 months helping to overcome diagnostic delay in Spain. - High sensitivity and specificity values. - Highlights the utility of using specific flowchart forms for each item during the phone interview. - Follow-up of false negatives with a monitoring system based on early intervention units in the education and welfare system. - The study has identified a clear need for coordination between the health services and ASD-specific early intervention unit in Spain.	- M-CHAT administration problems such as items being misunderstood and difficulties in locating families by telephone to apply the telephone interview. - Low positive predictive value. - No follow-up of negatives screen cases to confirm true negative cases. - Hard to demonstrate that ASD early-detection program using M-CHAT within a population-based framework is cost-effective to be implemented in the SNHS.
Nygren et al., 2012 [19]	- Health care professionals have learnt to use the instruments beyond the routine screening, increasing awareness and skills for recognition of symptomatology in ASD. - The study has notably improved the early diagnosis and intervention for children with autism by combining the screening methods with new routines for evaluation. In 2005, only 2 children of less than 3 years were referred and diagnosed, in 2010 the referred number was 48. - The screening programme described in the study is currently established as part of the developmental programme in the Child Health Centres.	- Attritions rates for diverse reasons at different stages of the programme: parents rejecting further evaluations, moving abroad, etc. - Psychometrical properties of the screening programme would not be possible to estimate with precision until long-term follow-up studies have been performed. - The given setting at 2.5-years-old visit would probably have been optimal half a year earlier.
Stenberg et al., 2014 [21]	- The prospective data collection and the combination of screening, referrals and registry linkage to identify ASD cases in the study sample, including children with mild and severe ASD symptoms, has allowed to better deal with the typical problems from the large population-based cohort design: rate of participation, losses in follow-up and selection bias. - Diagnosis reported at a very stable age (3 years).	- Ascertainment of ASD cases in the cohort is not yet completed. - Incomplete data is likely to have led to an overestimation of the psychometrical properties. - Follow-up interviews for positive screen cases in the M-CHAT is infeasible in a large population-based study like MoBa, with the consequence of a high rate of false positives. - The M-CHAT test translation applied was not culturally adapted. - The study could not assess the impact of social and cultural factors, such as parental awareness of early developmental markers associated with ASD and whether timing and composition of screening instruments can be optimised for maximal public health impact.
Baduel et al., 2017 [10]	- First ASD screening study in a French general population sample, showing the utility of the M-CHAT in primary-care settings. - The M-CHAT and the follow-up interview for positives cases in the questionnaire shows a large decrease in the rate of false positives and although some false positive cases remain, all cases presented developmental concerns, suggesting the use of M-CHAT is beneficial not just for children with ASD.	- Low number of children screened at 24 months due to low participation of primary care providers. - High rate of attrition on the follow-ups at 30 (70%) and 36 (35%) months to assure identification of false negative cases. - Given the high proportion of children with ASD found in this study, it is likely that paediatricians did not administer the M-CHAT on a routine basis to every child who came to their office but used it as a confirmation test when they had concerns. This underlines the importance of emphasizing that ASD screening should be systematic in paediatrician practice.
